# Matrix metalloproteinase-9 in relation to patients with complications after colorectal surgery: a systematic review

**DOI:** 10.1007/s00384-020-03724-6

**Published:** 2020-08-31

**Authors:** Pim Edomskis, Max R. Goudberg, Cloë L. Sparreboom, Anand G. Menon, Albert M. Wolthuis, Andre D’Hoore, Johan F. Lange

**Affiliations:** 1 Department of Surgery, Erasmus University Medical Center, Rotterdam, The Netherlands; 2 Department of Surgery, IJsselland Ziekenhuis, Capelle aan den IJssel, The Netherlands; 3 Departmenf of Abdominal Surgery, University Hospital Leuven, Leuven, Belgium

**Keywords:** Anastomotic leakage, Matrix metalloproteinase, Colorectal surgery, Complications

## Abstract

**Purpose:**

Anastomotic leakage (AL) is the most severe complication following colorectal resection and is associated with increased mortality. The main group of enzymes responsible for collagen and protein degradation in the extracellular matrix is matrix metalloproteinases. The literature is conflicting regarding anastomotic leakage and the degradation of extracellular collagen by matrix metalloproteinase-9 (MMP-9). In this systematic review, the possible correlation between anastomotic leakage after colorectal surgery and MMP-9 activity is investigated.

**Methods:**

Embase, MEDLINE, Cochrane, and Web of Science databases were searched up to 3 February 2020. All published articles that reported on the relationship between MMP-9 and anastomotic leakage were selected. Both human and animal studies were found eligible. The correlation between MMP-9 expression and anastomotic leakage after colorectal surgery.

**Results:**

Seven human studies and five animal studies were included for analysis. The human studies were subdivided into those assessing MMP-9 in peritoneal drain fluid, intestinal biopsies, and blood samples. Five out of seven human studies reported elevated levels of MMP-9 in patients with anastomotic leakage on different postoperative moments. The animal studies demonstrated that MMP-9 activity was highest in the direct vicinity of an anastomosis. Moreover, MMP-9 activity was significantly reduced in areas further proximally and distally from the anastomosis and was nearly or completely absent in uninjured tissue.

**Conclusion:**

Current literature shows some relation between MMP-9 activity and colorectal AL, but the evidence is inconsistent. Innovative techniques should further investigate the value of MMP-9 as a clinical biomarker for early detection, prevention, or treatment of AL.

**Electronic supplementary material:**

The online version of this article (10.1007/s00384-020-03724-6) contains supplementary material, which is available to authorized users.

## Introduction

Anastomotic leakage (AL) is the most feared complication following colorectal surgery and is associated with increased short- and long-term morbidity and mortality [1]. The reported incidence of AL varies between 6 and 8% after colon resection and between 7 and 20% after rectal resection [1–5]. Mortality rates related to AL range from 15.6 to 16.4% and 5.7 to 9.9%, for colonic and rectal resection, respectively [6–9]. Despite extensive research and ongoing advances in colorectal surgery, the incidence rates of AL have not declined over the last decades [10].

The current golden standard, C-reactive protein (CRP), has low-positive predictive accuracy for diagnosing AL [11]. Computer tomography is often added to increase the positive predictive value, but is not accurate enough to provide assurance of anastomotic integrity [12]. Early diagnosis of AL is of paramount importance because AL can result in higher morbidity and mortality [13]. The pathophysiology of AL seems multifactorial.

One potential element in the development of AL is degradation of the extracellular matrix (ECM), which in healthy tissue provides integrity and structure of intestinal tissue. Degradation of extracellular collagen is necessary after tissue injury, i.e., after surgery, in order to replace damaged tissue with healthy tissue to support healing. However, when balance is disturbed and an excess of collagen is degraded in proportion to newly produced collagen, this might lead to loss of integrity of an anastomosis and consequently to AL. Previously, experimental studies investigated the association between matrix metalloproteinases (MMPs) and AL and concluded that MMPs negatively affect anastomotic healing [14, 15]. Moreover, MMP inhibitors showed to enhance the breaking strength of colonic anastomoses during the early postoperative phase [16].

Specifically, matrix metalloproteinase-9, a zinc-dependent enzyme, plays a role in degradation of especially the collagen of which ECM is composed [17]. Until to date, it remains unknown whether this association represents a causal relationship or is a consequential effect of AL. In this systematic review, the association between MMP-9 and AL after colorectal surgery is assessed.

## Materials and methods

This study was conducted in accordance with the guidelines in the Preferred Reporting Items for Systematic Reviews and Meta-Analyses (PRISMA) statement [18]. Review Manager version 5.3 was used to perform statistical analyses. The protocol of this study was registered at the PROSPERO International prospective register of systematic reviews (ID-number: CRD140350).

### Systematic literature search

A systematic search was conducted with the assistance of a biomedical information specialist for studies assessing the association of MMP-9 activity and anastomotic healing after colorectal surgery. The Embase, MEDLINE, Cochrane, and Web of Science databases were searched from inception to January 24, 2019. An update of the search was performed on February 3, 2020. Full search syntaxes and results per database are shown in appendix 1.

### Study selection

Two researchers (MG, PE) independently reviewed the identified articles by title and abstract. Subsequently, full-text review was performed by two researchers (MG, CS) using EndNote X9® for Windows (Clarivate Analytics, Philadelphia, USA). Differences in article selection between reviewers were resolved through consensus by a third researcher (PE). Exclusion criteria included no full text available, article not published in a peer-reviewed journal, article language other than English, case reports, review articles, meta-analysis, letters, abstracts, or comments.

### Data extraction

Data extraction was performed by one researcher (MG) and validated by two other researchers (PE, CS). The following study details were collected: author, year of publication, country of origin, study design, number of participating centers, study length and length of follow-up, sample size, primary endpoint, MMP-9 measurement method, MMP-9 measurement moment, number of ALs, definition of AL, patient characteristics (sex, age, BMI for human studies, weight for animal studies.), and operative characteristics (type of surgery, surgical technique, hand-sewn or stapled anastomotic construction, operative time).

Included studies were categorized into two subgroups: human and animal studies. Human studies were further divided by type of measurement (e.g. peritoneal drain fluid, blood, or intestinal tissue) and animal studies were further divided by study outcome (e.g., studies assessing MMP-9 activity in relation to AL or studies assessing MMP-9 activity at different sites around a colorectal anastomosis). Discrepancies were discussed among all three researchers until consensus was reached. In case of uncertainties with regard to reported outcomes, corresponding authors were contacted when possible.

### Quality assessment

Study quality was assessed independently by two researchers (MG, PE) using the Newcastle-Ottawa Scale (NOS) and Methodological Index for Non-Randomized Studies (MINORS) criteria [19, 20]. For animal studies, the Systematic Review Centre for Laboratory Animal Experimentation (SYRCLE) tool was used [21]. Discrepancies in quality assessment outcomes were resolved by discussion between researchers.

## Results

### Systematic literature search

**Fig. 1:**
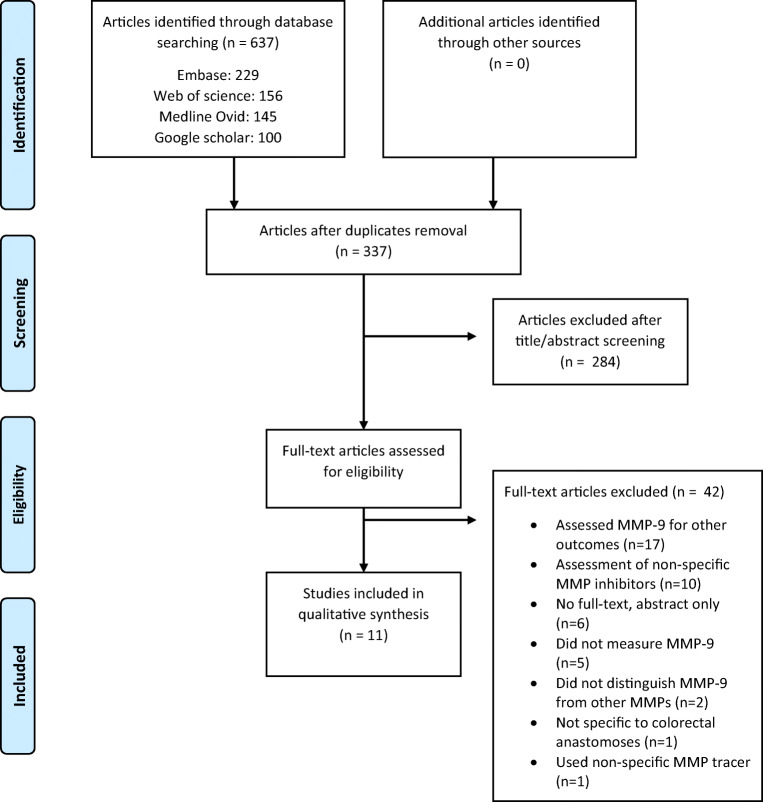
Preferred Reporting Items for Systematic Reviews and Meta-Analyses flow diagram of the study selection. MMP matrix metalloproteinase

Details of the study selection are provided in a PRISMA flow diagram (Fig. 1). The systematic literature search yielded 637 articles. After title and abstract screening, 53 articles remained for full-text review. A total of 12 articles were found eligible for inclusion after full-text reading.

### Study characteristics and data extraction

Seven human studies were included as well as five animal studies. Study characteristics for human and animal studies are summarized in Tables 1 and 2, respectively. All seven included human studies were prospective cohort studies of which one with matched controls. Data on a total of 785 patients who underwent colorectal resection and creation of an anastomosis was available. The human studies were subdivided into those assessing MMP-9 in peritoneal drain fluid, in intestinal biopsies, and in blood samples. Five studies used AL as primary endpoint. One study included AL in a group of “major complications.” One study included AL in an infection group, consisted of AL and intra-abdominal abscesses.

**Table 1:** Study characteristics of human studies with regard to anastomotic leakage

Author	Year	Study design	Level of evidence	Operations	Definition of anastomotic leakage	% anastomotic leakage	Method of measurement	Time of measurement	Main results
Drainage fluid									
Baker	2005	Prospective cohort	3	LAR; APR; sigmoidectomy; LHC; RHC; HP	Not reported	Not specified	Gelatinase activity assays	Daily until drain removed (POD 1–8)	MMP-9 significantly positively correlated with complications and their severity on POD 6 and 7.
Kostić	2003	Prospective cohort	3	Not reported	Clinical presence of purulent or fecal content at the drain site, pelvic abscess, peritonitis, rectovaginal fistula, or appearance of purulent content from the rectum.	10	ELISA	POD 1, 3, 5 and 7	No significant differences in MMP-9 levels between those with or without leakage on POD 1, 3, 5, or 7.
Pasternak	2015	Prospective cohort	3	LAR	Clinical: peritonitis caused by leakage, discharge of feces from the abdominal drain, rectovaginal fistula, leakage from all the staple lines, pelvic abscess without radiologically proven leakage mechanism. Confirmed by either rectal contrast radiology, CT, rigid rectoscopy, flexible sigmoidoscopy, or digital rectal palpation.	34	Particle-based multiplex flow-cytometry	Median at 4 h postoperative (range 2–6 h)	Significant higher levels of MMP-9 were found in patients who later developed anastomotic leakage
Sparreboom	2010	Prospective cohort	3	PME; TME	Clinically manifest insufficiency of the anastomosis leading to a clinical state requiring treatment. Confirmed by either endoscopy, CT scan, and/or contrast enema or reoperation.	13	Immunoassay	POD 1,2, and 3	Significantly higher MMP-9 levels on POD 3 in those with anastomotic leakage
Intestinal biopsies									
Angenete	2007	Prospective cohort	3	LAR; APR	Clinically verified anastomotic dehiscence.	2	ELISA	During surgery	No correlation between MMP-9 and anastomotic leakage
Stumpf	2019	Prospective cohort	3	Not reported	Clinically presence of a fecal fistula or fecal fluid over the drainage. Confirmed by endoscopy or radiologic enema.	13	Immunoreactive score	During surgery	Significant higher MMP-9 scores in the submucosal layers of patients with anastomotic leakage compared with those without
Blood samples									
Alonso	2017	Prospective cohort with matched controls	3	Not reported	Clinical suspicion confirmed with radiological or operative exploration.	6	Gene expression and RT-PCR	POD 4 (or in case of intra-abdominal infection)	Significant more upregulated genes coding for MMP-9 in patients in with anastomotic leakage

*HP*, Hartmann’s procedure; *PME*, partial mesorectal excision; *TME*, total mesorectal excision; *LAR*, low anterior resection; *APR*, abdominoperineal resection; *LHC*, left hemicolectomy; *RHC*, right hemicolectomy; *MMP-9*, matrix metalloproteinase-9; *POD*, postoperative day; *CT*, computed tomography; *RT-PCR*, real time-polymerase chain reaction; *ELISA*, enzyme-linked immunosorbent assay

**Table 2:** Study characteristics of animal studies in relation to anastomotic leakage

Author	Year	Level of evidence	Operations	Definition of anastomotic leakage	Method of measurement	Time of measurement	Main results
MMP-9 in relation to AL							
Shogan	2015	5	Distal colon segment resection	Visible dense adhesions to the anastomotic line indicating inflammation; a hard, grossly inflamed and non-compressible anastomosis; grossly disrupted anastomotic site with adjacent purulent like material or a grossly visible hole and ulceration and disruption on the luminal mucosal side.	Zymography and Western blot	POD 6	1. Significantly higher MMP-9 activity in rats with anastomotic leakage 2. MMP-9 inhibition results in lower leakage rates
MMP-9 at different sites around anastomosis							
Ågren	2006	5	Distal colon segment resection	Not applicable	Immunohistochemistry	Day 0, POD 3 or 7	1. No MMP-9 activity on day 0. 2. More MMP-9 positive cells in the anastomotis area compared with uninjured areas. 3. Higher MMP-9 levels directly around the sutures compared with suture-free zones of the anastomotis area.
De Hingh	2005	5	Distal colon transection	Not applicable	Zymography	Day 0 (no intervention), POD 1 or 3	Significantly higher MMP-9 activity on POD 1 and 3 in the anastomosis area compared with uninjured intestine.
Seifert	1996	5	Distal colon segment resection	Not applicable	Zymography	Day 0, POD 1, 2, 3, 7, or 90	1. No MMP-9 activity on day 0 and POD 90 2. Higher MMP-9 activity in anastomosis area on POD 1, 2, 3, and 7 compared with uninjured areas.
Shaper	2001	5	Distal colon segment resection	Not applicable	Zymography and Immunohistochemistry	6 h after surgery or POD 7	More MMP-9 activity in anastomosis area at 6 h postoperative and on POD 7 compared with uninjured areas.

*MMP-9*, matrix metalloproteinase-9; *POD*, postoperative day

The animal studies were subdivided into those assessing MMP-9 activity in relation to AL and those assessing MMP-9 activity at different sites around the anastomosis. Four studies assessed MMP-9 activity at different sites around the anastomosis and one study assessed MMP-9 activity in relation to AL.

### Definition and rates of anastomotic leakage

The included studies diagnosed AL mostly by clinical suspicion confirmed by radiological or operative exploration. Incidence of AL in the studies ranged from 1.1 to 34.5% with a mean AL rate of 10.8%.

### Quality assessment

**Fig. 2:**
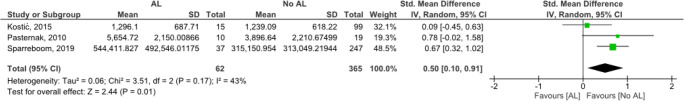
Forest plot of cohort studies comparing anastomotic leakage and non-anastomotic leakage on MMP-9 activity in peritoneal drain fluid on postoperative day 1

**Fig. 3:**
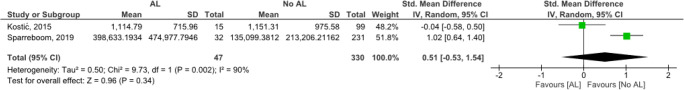
Forest plot of cohort studies comparing anastomotic leakage and non-anastomotic leakage on MMP-9 activity in peritoneal drain fluid on postoperative day 3

Results from the quality assessment with the NOS, MINORS instruments, and the SYRCLE tool are shown in Tables S1, S2 and S3, respectively. The MINORS instrument scored four studies as of moderate quality and three of poor quality. The degree of statistical heterogeneity among studies was estimated by calculating *I*^2^, as shown in Figs. 2 and 3, respectively. The risk of bias assessed with the SYRCLE tool in the animal studies was overall unclear or low. Three of the five animal studies had high risk of bias on the item “sequence generation” for not describing if the allocation to groups was adequately generated and applied. No study described the concealment of allocation, random housing, and blinding of caretakers and therefore, the risk of bias for these items was unclear for all studies. On all other items, the studies had low risk of bias.

### Human studies

#### Peritoneal drain fluid

Four studies assessed peritoneal drain fluid. Baker et al. included 58 patients who underwent colorectal resection (e.g., right hemicolectomy, left hemicolectomy, sigmoid colectomy, low anterior resection, or Hartmann’s procedure) [22]. MMP-9 was measured daily in peritoneal drain fluid using gelatinase activity assays until the drain was removed. The number of ALs was not specified from a group of 16 patients having major complications, but there was a significant positive correlation between MMP-9 and postoperative complications and their severity on postoperative day (POD) 6 and 7. No absolute values or confidence intervals were reported [22].

Data of three other studies assessing peritoneal drain fluid could be pooled for outcomes on POD 1. As shown in Fig. 2, there was a significant difference in mean MMP-9 expression in patients with and without anastomotic leakage. Two studies assessing peritoneal drain fluid on POD 3 could also be compared but differences in mean MMP-9 expression were not significant (Fig. 3).

Kostić et al. measured MMP-9 activity in peritoneal drain fluid from 150 patients using ELISA on day 1, 3, 5, and 7 postoperatively [23]. All patients underwent left-sided colorectal resections. Of these patients, 15 (10%) developed AL. No significant differences were observed in mean levels of MMP-9 for patients with and without AL [23].

Pasternak et al. conducted a study in which MMP-9 levels of 29 patients were measured in peritoneal drain fluid using a particle-based multiplex flow-cytometry at a median of 4 h (range 2–6 h) after low anterior resection [24]. Ten patients (34.5%) developed AL. Significant higher levels of MMP-9 were found in patients who later developed AL with a median difference of 1180 ng/ml (95% CI 141–3050, *p* = 0.03) [24].

The study of Sparreboom et al. analyzed 292 patients who underwent partial mesorectal excision or total mesorectal excision [25]. MMP-9 levels in peritoneal drain fluid were measured on POD 1, 2, and 3 using immunoassays. A total of 38 patients (13%) developed AL. They found significant higher levels of MMP-9 in patients with AL compared with patients without AL on POD 3 (2.0 × 10^5^ pg/mL IQR 0.5–5.0 vs. 0.6 × 10^5^ pg/mL IQR 0.3–1.5, *p* = 0.011). On POD 1 and 2, the MMP-9 levels in patients with AL were also higher; however, these differences were statistically not significant. In addition, this study showed that the combination of serum CRP and peritoneal MMP-9 on POD 3 was predictive for AL (AUC =0.78) [25].

#### Intestinal tissue

Two studies measured MMP-9 in intestinal tissue samples. Angenete et al. measured MMP-9 in 61 patients using ELISA on biopsies taken from the resected rectal segment, which was snap frozen in liquid nitrogen during surgery [26]. These patients underwent low anterior resection, only one of 61 patients (1.6%) developed AL and no correlation with MMP-9 was found [26]. Stumpf et al. used an immunoreactive score to measure MMP-9 activity in biopsies taken from the resected colorectal segment during surgery, which were paraffin-embedded [27]. This score ranged from 1 with low amounts of MMP-9 positive cells to 20 with high amounts of MMP-9 positive cells. In 119 patients, a colorectal resection was performed in which 15 (12.6%) of them developed AL. Type of surgical resection was not further specified. Significant higher MMP-9 scores were found in the submucosal layers of patients with AL compared with those without AL (median = 13, IQR (11–14) vs. median = 11, IQR (9–13) (*p* < 0.05)) [27]. These scores were measured manually from figures in the article, since exact data could not be retrieved after consultation of the author.

#### Blood samples

In a cohort of 340 patients, Alonso et al. investigated expression of genes coding for MMP-9 in peripheral blood leucocytes using microarray expression profiles from blood samples obtained 4 days after surgery, or earlier in case AL was diagnosed [28]. Validation of the expression profiles was done with RT-PCR. The patients underwent colorectal surgery without further characterization. In this cohort, 21 patients (6.2%) developed AL, 23 patients (6.8%) acquired an infection, and 2 (0.6%) patients were diagnosed with an intra-abdominal abscess. These patients were matched for gender, age, tumor location, surgical approach, date of operation, and tumor stage according to the TNM classification with healthy controls who had an uncomplicated postoperative course. Genes coding for MMP-9 were significantly upregulated in the infection group compared with their controls (4.9-fold change, *p* < 0.001). RT-PCR validation also found significant higher MMP-9 mRNA levels in patients in the infection group (*p* = 0.001) [28].

### Animal studies

#### MMP-9 in relation to AL

The study of Shogan et al. measured MMP-9 activity in rats using zymography and western blots [29]. Fifteen rats underwent a colon segment resection of 1 cm at the peritoneal reflection with devascularization. MMP-9 activity was higher in extracted tissue of rats with AL compared with rats with healed anastomotic tissue. Pharmacologic inhibition of MMP-9 resulted in suppressed MMP-9 and lower AL rates [29]. In this study, the number of rats developing AL was not specified and absolute values or confidence intervals were not reported.

#### MMP-9 at different sites around anastomosis

Four studies assessed MMP-9 at different sites around the anastomosis. Ågren et al. analyzed MMP-9 activity in rats using immunohistochemistry on POD 0, 3, or 7 [30]. Measurement was done with resection of a 3-cm segment where the anastomosis was centrally located. No MMP-9 reactivity was found at any site on POD 0. On POD 3 and 7, more MMP-9 positive cells were found in the anastomotic wound area compared with the adjacent uninjured areas proximally and distally from the anastomosis. Moreover, the MMP-9 levels directly around the sutures were higher compared with the suture-free zone of the anastomotic wound area [30].

De Hingh et al. used zymography on POD 0 (no intervention), 1, or 3 to measure MMP-9 activity in 12 rats [31]. Six rats were included in the control group; this group was sacrificed without any intervention. From the other six rats, the distal colon was transected, and an anastomosis was created. On POD 1 or 3, a 2.5-cm segment with the anastomosis was resected. In the uninjured colon of the control group, MMP-9 activity could not be found. Furthermore, significant higher MMP-9 activity was found on POD 1 and 3 in the area of the anastomosis compared with uninjured colon proximally and distally from the anastomosis (*p* < 0.001) [31].

Seifert et al. investigated MMP-9 activity measured with zymography in 18 rats [32]. Three rats were included in the control group and were sacrificed without undergoing any operation. The other 15 rats underwent a 1-cm colon segment resection and were sacrificed on POD 1, 2, 3, 7, or 90, after which a 0.5-cm segment together with the anastomosis was resected. No MMP-9 activity was found in the uninjured control group or on POD 90. At other time points, measured MMP-9 activity was significantly higher directly around the anastomosis compared with uninjured tissue [32].

Shaper et al. measured MMP-9 activity in 40 rabbits using immunohistochemistry and zymography [33]. A distal 0.5-cm segment of the colon was resected and an anastomosis was created. The resected segment was used as control for analysis. Half of the rabbits were sacrificed on POD 0 and the other half on POD 7, after which a 1-cm segment with the anastomosis centralized was resected. MMP-9 was not detected by immunohistochemistry in the control group, but in some controls, MMP-9 activity was found with zymography. There was more MMP-9 activity around the anastomosis on POD 0 and 7 compared with the control segments and adjacent uninjured colon segments distal and proximal from the anastomosis.

In summary, in animal models, MMP-9 expression was higher in the area of anastomosis compared with uninjured tissue. Also, MMP-9 expression was highest near the anastomosis and decreased further away from it.

## Discussion

This systematic review shows that there is a connection between patients with AL or infectious complication and elevated levels of MMP-9 on different postoperative moments in five out of seven human studies [22, 24, 25, 27, 28]. Two other human studies did not find a significant difference in MMP-9 levels [23, 26]. These studies by Kostić et al. and Angenete et al. diagnosed AL only clinically, without radiological confirmation, which is in contrast with other studies. Although the value of radiological detection of AL is questionable, this could possibly explain the difference in outcome between the studies [34, 35]. In addition, in the study by Angenete et al., only one of 91 patients developed AL and the study by Baker et al. did not specify the amount or percentage of AL. Presumably this was caused by a heterogenous study population, that also included abdominoperineal resections. With such a small event size and heterogenous population, it is unlikely to detect a possible correlation.

A potential element in the development of AL is degradation of the extracellular matrix (ECM), which in healthy tissue provides integrity and structure of intestinal tissue. Degradation of extracellular collagen is necessary after tissue injury, i.e., after surgery, in order to replace damaged tissue with healthy tissue. Imbalances in collagen degradation are possibly caused by members of the intestinal commensal microflora. Shogan et al. showed that numbers of several Enterococcus species, including *Enterococcus faecalis*, were increased in anastomotic tissue after anastomosis construction [36]. Another study reported that *Enterococcus faecalis* has the capacity to degrade collagen and support MMP-9 activation [29]. Therefore, the increase of MMP-9 levels could play a role in early detection and in innovative treatment strategies to minimize the consequences of infection and AL in the near future.

Previous studies have assessed the influence of MMP inhibition, including MMP-9, on anastomotic healing. These studies showed that anastomotic breaking strength is increased after MMP inhibition [16, 37, 38]. However, no increase in collagen levels was found in the analyzed segments. It is suggested that collagen levels were not increased because only a small amount of tissue directly adjacent to the sutures and anastomosis was involved. By analyzing the whole anastomotic segment, including adjacent non-involved tissue, increase of collagen might be too subtle to detect [37].

The animal study that assessed the relation between MMP-9 and AL also showed higher MMP-9 activity in intestinal tissue in animals with AL [29]. Furthermore, our subgroup of animal studies assessing MMP-9 at different sites around the anastomosis was consistent in their findings. These studies showed that MMP-9 activity was highest directly around an anastomosis. Also, MMP-9 activity was significantly reduced in areas adjacent proximally and distally from the anastomosis and was nearly or completely absent in uninjured tissue. This confirms that loss of anastomotic strength due to MMP-9 activity and loss of collagen is a very localized process.

Previous systematic reviews have investigated the role of several biomarkers in the prediction and diagnosis of colorectal AL, including MMP-9. Cini et al. concluded in 2013 in a review of two studies assessing MMP-9 that patients with AL had higher levels of peritoneal MMP-9 in drain fluid than patients without AL [39]. On the other hand, two reviews from 2017 by Su’a et al. and Wright et al. had inconsistent or no conclusions on the role of MMP-9 in the diagnosis and detection of colorectal AL [40, 41].

There are some limitations to the present study that are important to acknowledge. At first, there was substantial methodological heterogeneity between included studies. In particular there was a variance in measurement, both frequency and specific days of MMP-9 measurement were reasons for incomparability of the peritoneal drain fluid studies. Therefore, pooling data for a meta-analysis was impossible. The definition for AL too varied between the studies, even in studies published after the proposed grading system by the International Study Group of Rectal Cancer [42]. Furthermore, the included studies were all observational or experimental and were classified as of low or moderate quality. Moreover, this systematic review also included animal studies to collect all possible evidence with regard to MMP-9 and anastomotic leakage. Conclusions drawn from these studies should be handled delicately since findings are not always transferable to human physiology.

This is the first review to report on the association between MMP-9 activity and anastomotic leakage. Understanding the pathophysiology to be able to detect AL in an early stage is of paramount importance in daily clinical practice. It is of great clinical value to investigate whether MMP-9 could function as a biomarker for AL or as an innovative strategy for prevention and treatment via specific MMP inhibitors. The study of Sparreboom et al. already showed the predictive value of MMP-9 in addition to CRP with regard to AL. Future research should focus on a standardized method and a high-quality prospective study to assess the role of MMP-9 in the development of AL after colorectal surgery. New sensors and measurement methods are warranted to clarify the potential of MMP-9 as a clinical biomarker.

In conclusion**,** current literature shows some relation between MMP-9 activity and colorectal AL, but current evidence is inconsistent. Innovative techniques should further investigate the value of MMP-9 as a clinical biomarker for early detection, prevention, or treatment of AL.

## Electronic supplementary material

ESM 1 (DOCX 14 kb)

ESM 2 (XLSX 12 kb)

ESM 3 (XLSX 11 kb)

ESM 4 (XLSX 12 kb)

## Data Availability

Available upon request.
