# Supplementary material for: Matrix metalloproteinase-9 in relation to patients with complications after colorectal surgery: a systematic review
Source: Int J Colorectal Dis. 2020 Aug 31;36(1):1–10. doi: 10.1007/s00384-020-03724-6 (PMC7782374; doi:10.1007/s00384-020-03724-6)
Supplement: Supplementary file 1 — (DOCX 14 kb) [file 384_2020_3724_MOESM1_ESM.docx]

**Appendix S1**

Full search syntaxes and results per database.

**Embase.com:** 229 articles

('metalloproteinase'/exp OR 'metalloproteinase inhibitor'/exp OR ('extracellular matrix'/de AND (proteinase/exp OR 'proteinase inhibitor'/exp)) OR (metalloprote* OR matrixmetalloprote* OR matrixmetallo-prote* OR metallo-prote* OR mmp* OR collagenase* OR gelatinase* OR macrophage-elastase* OR matrilysin OR stromelysin*):ab,ti,kw) AND ('anastomosis leakage'/de OR 'anastomosis dehiscence'/de OR (anastomosis/exp AND (rupture/de OR 'wound dehiscence'/de OR 'wound healing'/de OR healing/de OR 'tensile strength'/de OR 'wound healing impairment'/de)) OR ((anastomo* AND (leak* OR healing OR dehiscence* OR wound OR strength OR rupture* OR break*))):ab,ti,kw)

**Medline Ovid:** 145 articles

(exp Metalloproteases/ OR exp Tissue Inhibitor of Metalloproteinases/ OR exp Matrix Metalloproteinase Inhibitors/ OR (Extracellular Matrix/ AND (proteinase/ OR Protease Inhibitors/)) OR (metalloprote* OR matrixmetalloprote* OR matrixmetallo-prote* OR metallo-prote* OR mmp* OR collagenase* OR gelatinase* OR macrophage-elastase* OR matrilysin OR stromelysin*).ab,ti,kw.) AND (Anastomotic Leak/ OR (exp Anastomosis, Surgical/ AND (Rupture/ OR Surgical Wound Dehiscence/ OR Wound Healing/ OR Tensile Strength/)) OR ((anastomo* AND (leak* OR healing OR dehiscence* OR wound OR strength OR rupture* OR break*))).ab,ti,kw.)

**Web of science:** 156 articles

TS=(((metalloprote* OR matrixmetalloprote* OR matrixmetallo-prote* OR metallo-prote* OR mmp* OR collagenase* OR gelatinase* OR macrophage-elastase* OR matrilysin OR stromelysin*)) AND (((anastomo* AND (leak* OR healing OR dehiscence* OR wound OR strength OR rupture* OR break*)))))

**Cochrane CENTRAL:** 7 articles

((metalloprote* OR matrixmetalloprote* OR matrixmetallo-prote* OR metallo-prote* OR mmp* OR collagenase* OR gelatinase* OR macrophage-elastase* OR matrilysin OR stromelysin*):ab,ti,kw) AND (((anastomo* AND (leak* OR healing OR dehiscence* OR wound OR strength OR rupture* OR break*))):ab,ti,kw)

**Google scholar:** 100 articles

metalloproteinase|matrixmetalloproteinase|collagenase|gelatinase|"macrophage-elastase"|matrilysin|stromelysin anastomotic|anastomosis leak|leakage|healing|dehiscence|wound|strength|rupture|break
